# Pharmacogenetic Considerations in Sertraline Augmentation With Quetiapine in an Adolescent Woman With Obsessive-Compulsive Disorder, Autism Spectrum Disorder, and Attention-Deficit Hyperactivity Disorder: A Case Report

**DOI:** 10.7759/cureus.102813

**Published:** 2026-02-02

**Authors:** Adebusola Adegbola, Nikhil Dhir, Omotola Ogunjobi

**Affiliations:** 1 Psychiatry, Humber Teaching National Health Service (NHS) Foundation Trust, Kingston upon Hull, GBR; 2 Psychiatry, South West London and St George's Mental Health National Health Service (NHS) Trust, London, GBR; 3 Community Mental Health Services, Black Country Healthcare Mental Health Trust, Dudley, GBR

**Keywords:** antipsychotic augmentation, autism spectrum disorder (asd), obsessive compulsive disorders, pharmacogenetic testing, quetiapine augmentation

## Abstract

Obsessive compulsive disorder (OCD) in children and young people commonly presents alongside neurodevelopmental comorbidities such as autism spectrum disorder (ASD) and attention-deficit hyperactivity disorder (ADHD). These presentations are often associated with greater functional impairment and pose significant diagnostic and treatment challenges. We describe the case of an adolescent woman with OCD, ASD, and ADHD who presented with a deterioration in her mental state, characterised by symmetry-related grooming and self-care rituals lasting up to four hours daily, low mood, poor sleep, social withdrawal, suicidal ideation, and marked impairment in daily functioning. She had previously been stabilised on sertraline (200 mg) and lisdexamfetamine (60 mg). Worsening symptoms and functional decline raised the possibility of benefit from antipsychotic augmentation, and quetiapine was initiated. This was guided by pharmacogenetic testing following a poor initial response to ADHD medication and adverse effects when sertraline was first commenced. After three months of treatment with quetiapine, she demonstrated functional improvement, including a reduction in symmetry rituals and improved social engagement. This case highlights the complexities of psychological and pharmacological management of OCD in the context of neurodevelopmental comorbidity. Improved understanding of precision psychiatry in OCD with ASD comorbidity may facilitate the development of more tailored treatment approaches, including neurodevelopmentally adapted psychological interventions and targeted pharmacological strategies.

## Introduction

Antipsychotic augmentation is recommended as an off-label treatment in children with treatment-resistant obsessive compulsive disorder (OCD) after at least a 12-week trial of two different selective serotonin reuptake inhibitors (SSRI) at the maximum tolerated dose [1,2]. Generally, risperidone and aripiprazole are the recommended options given their relative tolerability and side effect profiles [1,2]. In this case, antipsychotic augmentation was initiated after one trial of SSRI (sertraline) and the antipsychotic of choice was quetiapine. This was based on family preference following pharmacogenetic testing, making this case rather interesting.

## Case presentation

A 17-year-old adolescent with a known history of ASD, ADHD, low mood, anxiety, and OCD presented to the Child and Adolescent Mental Health Service (CAMHS) Tier 3 clinic due to a deterioration in her mental state. Tier 3 CAMHS is a multidisciplinary, specialist mental health, community-based service for moderate to severe mental health conditions, offering complex and targeted interventions. OCD was diagnosed in the previous year, and she subsequently underwent intensive psychological and pharmacological interventions within Tier 3 CAMHS and the Adolescent Outreach Team, a Tier 4 CAMHS service (highly specialist, inpatient or day-patient services including intensive home treatment for severe, complex, or life-threatening conditions where other services such as Tier 3 are not sufficient). She was unable to engage with cognitive behavioural therapy (CBT), and a referral was made to the Children and Young People’s OCD and body dysmorphic disorder (BDD) specialist service, where sertraline was titrated to 200 mg daily. She was also offered a 20-session package of CBT with exposure and response prevention (ERP) for OCD, adapted for ASD, of which she attended 11 sessions. During this period, she experienced difficulties with regular attendance and consistent engagement with therapy homework.

Despite these challenges, significant improvement was noted following the therapy sessions, and she was able to return to boarding school on sertraline 200 mg once daily for OCD and lisdexamfetamine 60 mg once in the morning for ADHD. After several months of relative stability, her residential school placement broke down due to symmetry rituals lasting up to four hours, leading to missed lessons, and the school was deemed unable to meet her needs. Other OCD themes included contamination and self-care rituals requiring things to feel “just right” before she was able to proceed.

She subsequently discontinued lisdexamfetamine since she was no longer in school but remained concordant with sertraline 200 mg daily. Alongside her parents, she reported a steady decline in her mood, avoidance of self-care to prevent self-care-related symmetry rituals (e.g. showering in a particular manner, brushing her hair and checking for loose strands), increasing social withdrawal with isolation in her room, sleep reversal, and pessimism about her future with passive suicidal ideation. She became largely housebound and had no daily structure due to the amount of time spent engaging in compulsive rituals.

Significant mental state examination findings at this presentation included low mood, dichotomous (black-and-white) thinking, pessimism, and suicidal ideation with no clear obsessive content.

Given the diagnostic complexity, the patient’s repetitive behaviours were conceptualised across obsessive-compulsive and autism-related domains, with some overlapping features (Table 1).

**Table 1:** Phenomenology of repetitive behaviours: OCD versus ASD in this case OCD: Obsessive compulsive disorder, ASD: autism spectrum disorder, SSRI: selective serotonin reuptake inhibitors, CBT: cognitive behavioural therapy, ERP: exposure and response prevention.

Domain	OCD-related compulsions	ASD repetitive behaviours	Overlapping features
Trigger	Obsession and compulsions (e.g. contamination and symmetry)	Sensory discomfort/predictability	“Just right” feeling
Function	To reduce anxiety from intrusive thoughts	Soothing and pleasurable for sensory modulation	Distress when interrupted
Flexibility	Rigid, resisted, ego-dystonic	More habitual, ego-syntonic	Repetition and insistence
Example in this case	Prolonged symmetry rituals delaying school attendance	Repetitive grooming linked to sensory sensitivity	Avoidance of self-care
Treatment response	Partial response to SSRI treatment	Difficulty engaging with structured CBT/ERP tasks	Limited response to ERP despite adaptation for ASD

**Figure 1:**
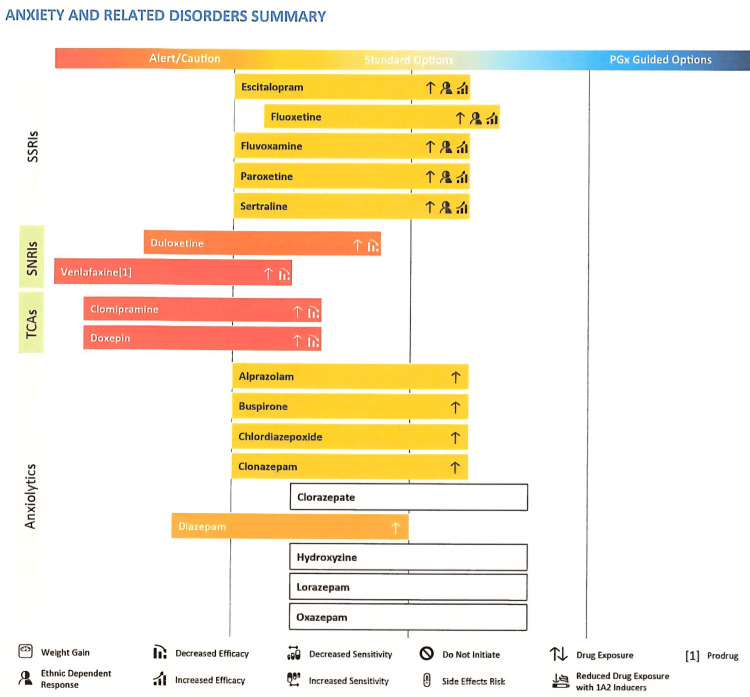
Pharmacogenetic test results for anxiety and related disorders. The genetic test provided guidance that the young person’s metabolism of all selective serotonin reuptake inhibitors (SSRIs) is reduced due to a variant in the gene involved in the metabolic pathway. This indicates that there is an increased exposure to SSRIs as well as ethnic-dependent response and increased efficacy, which may explain why she experienced side effects on sertraline at a therapeutic dose of 100 mg. The genetic test suggests increased exposure and reduced efficacy for serotonin noradrenaline reuptake inhibitors (SNRIs) like venlafaxine and duloxetine, and tricyclic antidepressants (TCAs) like clomipramine and doxepin as well as increased exposure for benzodiazepines as seen in diazepam, alprazolam, and clonazepam.

**Figure 2:**
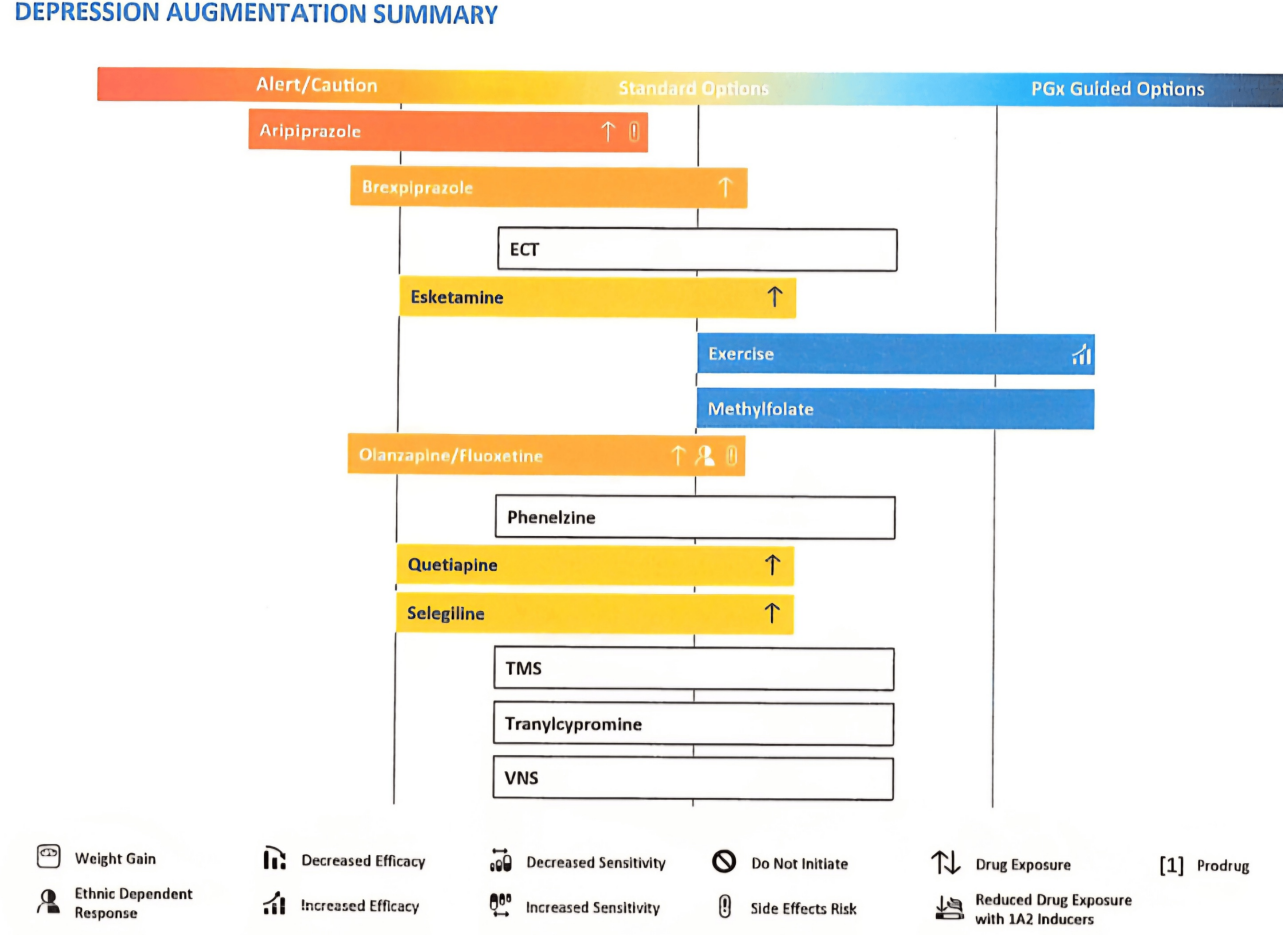
Pharmacogenetic test result for antipsychotic augmentation in depression Antipsychotic augmentation in depression suggests an increased risk of drug exposure. It appears to be even more so for aripiprazole in addition to an increased risk of side effects. This genetic test suggests that exercise and methylfolate supplementation may improve symptoms of depression for this adolescent. Methylenetetrahydrofolate reductase (MTHFR) is an enzyme responsible for converting folic acid to methylfolate, which is a co-factor for serotonin, norepinephrine, and dopamine synthesis [3]. Therefore, supplementing selective serotonin reuptake inhibitors (SSRIs) or serotonin noradrenaline reuptake inhibitors (SNRIs) may result in better depressive symptoms reduction. Olanzapine/fluoxetine combination also suggested increased drug exposure, ethnic dependent response, and increased risk of side effects. TMS: Transcranial magnetic stimulation; VNS: vagus nerve stimulation.

**Figure 3:**
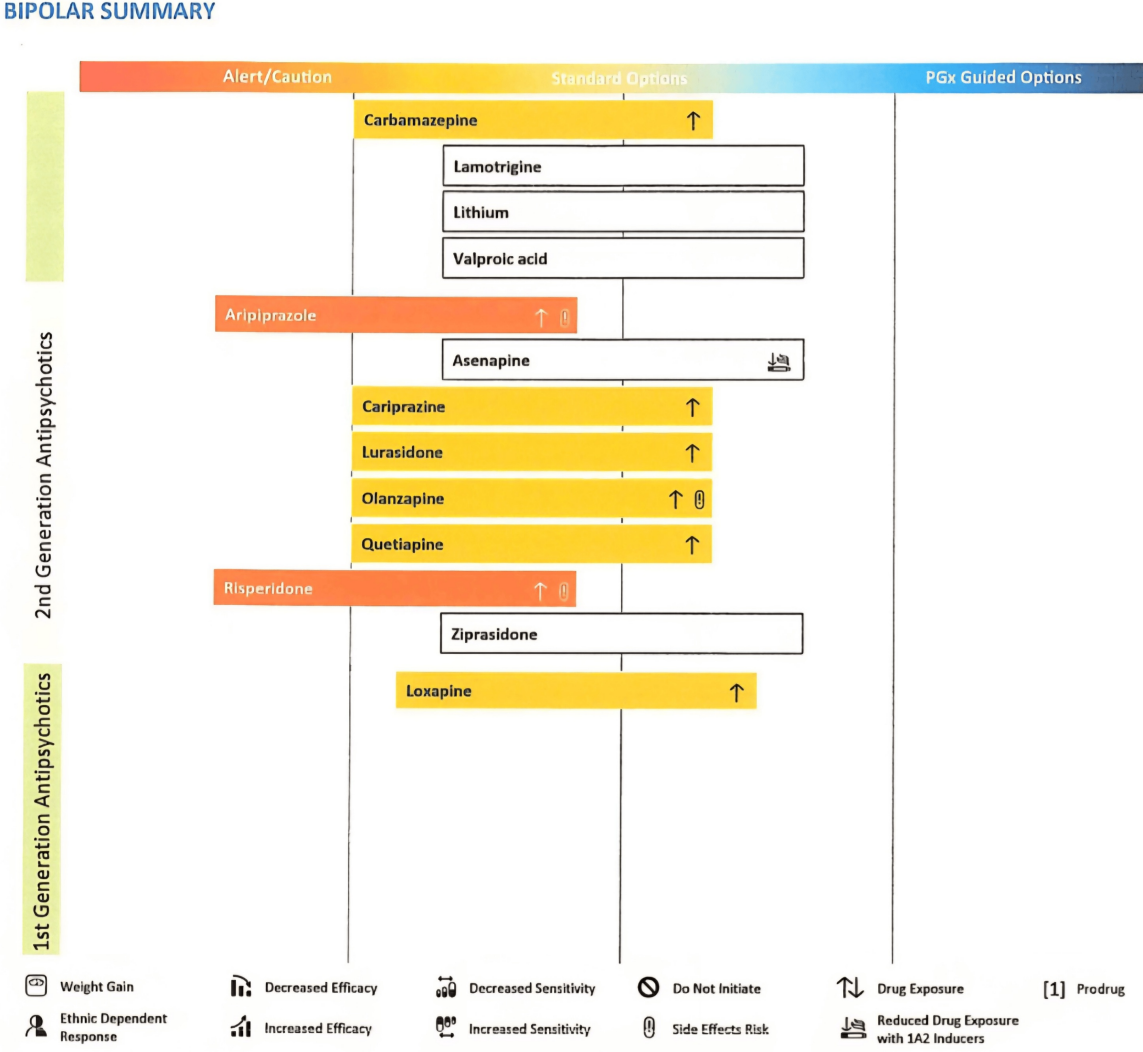
Pharmacogenetic test result for antipsychotic use in bipolar While we appreciate that the young person does not have bipolar, like aripiprazole, the genetic test suggests increased drug exposure and risk of side effects for risperidone.

**Figure 4:**
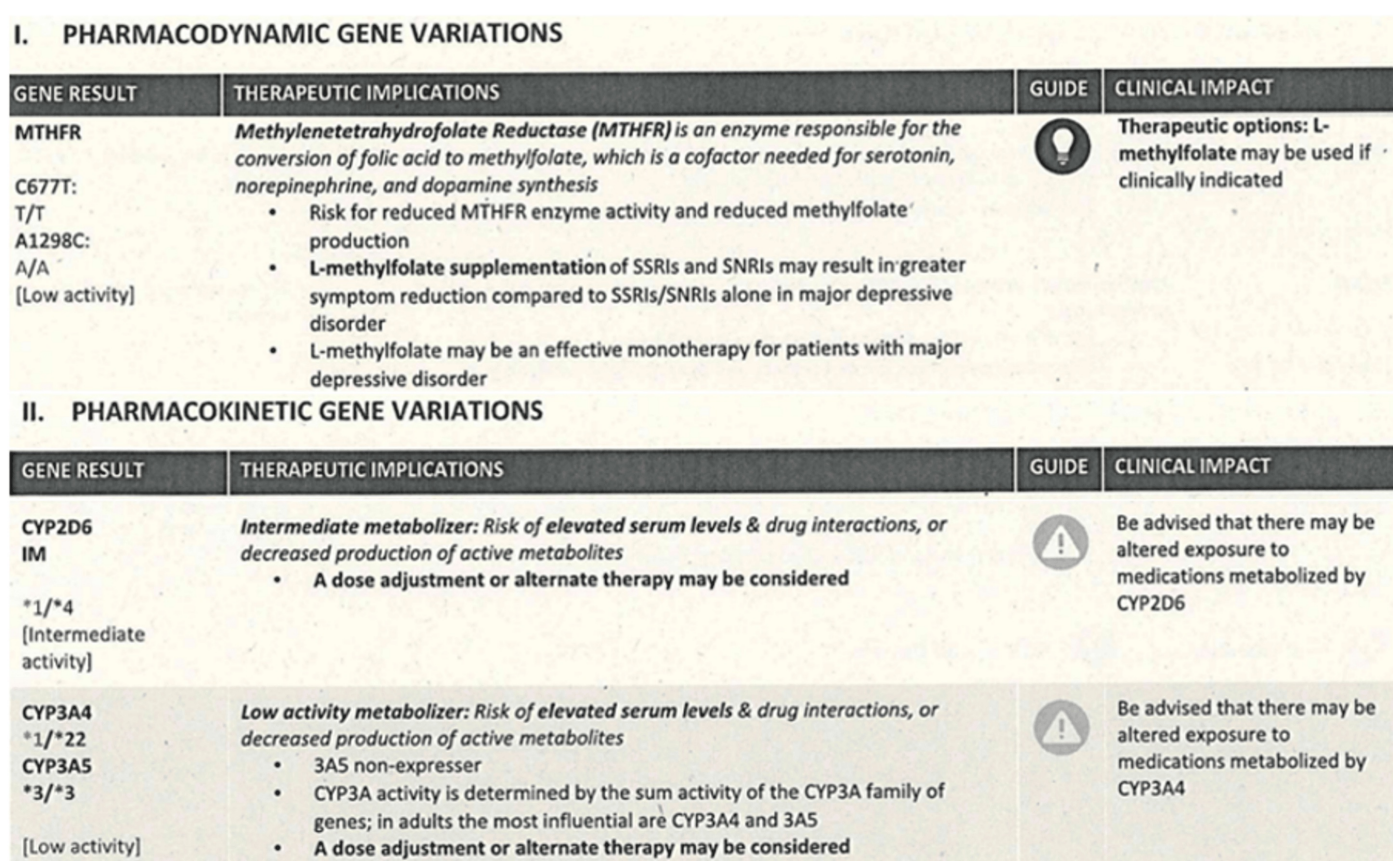
Pharmacogenetic gene variations relevant to this case This figure summarises the relevance of methylenetetrahydrofolate reductase (MTHFR) highlighted in Figure 2, cytochrome P450 2D6 (CYP2D6), and cytochrome P450 3A4 (CYP3A4) genetic variants to psychotropic prescribing. The patient was identified as an intermediate metaboliser of CYP2D6. She was also found to have low CYP3A4 activity. Risperidone and aripiprazole are metabolised significantly by CYP2D6 (with CYP3A4 playing a secondary role for both) while quetiapine is predominantly metabolised by CYP3A4 [4]. Variants resulting in intermediate or poor metabolism lead to elevated drug concentrations, longer half-life, and higher risk of side effects. Given the patient’s intermediate CYP2D6 status and low CYP3A4 activity, the family preferred quetiapine given its CYP3A4-predominant pathway with cautious dose titration to balance efficacy and tolerability.

Her past psychiatric history indicated that she had been known to mental health services since the age of seven, with diagnoses of ASD, ADHD, low mood, and anxiety. She was initially trialled on methylphenidate for ADHD; however, due to a poor response, this was switched to lisdexamfetamine, which resulted in improved focus and concentration. She was later commenced on sertraline for anxiety and low mood, which was initially poorly tolerated. It was reported that she exhibited aggressive and suicidal behaviours, which were severe enough to necessitate presentation to the accident and emergency department. This prompted pharmacogenetic testing (Figures 1-4) to gain a better understanding of how her genetic profile might influence medication metabolism and treatment response.

The patient and her family were offered a switch to another SSRI (fluoxetine) to help improve OCD and mood symptoms in line with National Institute of Health and Care Excellence (NICE) and Maudsley Prescribing Guidelines [1,2] but due to concerns about switching SSRIs and previous sensitivity to medications (including suicidal ideation when sertraline was initially increased), the family preferred not to switch to another SSRI. The adolescent clearly stated that sertraline has been helpful by providing partial relief as ‘it made difficult things easier’ and she and her family were hoping for augmentation with a low dose of antipsychotic medication instead. Augmentation strategies were discussed. While treatment guidelines [1,2] support the use of low-dose risperidone or aripiprazole for OCD augmentation, the family preferred quetiapine, a decision informed by the findings of the genetic testing and possible benefits for mood, poor sleep, and ASD-related symptoms. The family was made aware that while the pharma­cogenetic test is helpful for information around tolerability and risk of side effects, it is not definitive in predicting the efficacy of quetiapine.

Contact was made with the children and young people’s OCD and body dysmorphic disorder (BDD) specialist service for co-management. After adequate time and efforts to discuss therapeutic rationale and considering pros and cons, including the risk of metabolic concerns given her baseline physical investigations, she made an informed decision to try quetiapine 12.5mg twice daily with parental support.

Before starting quetiapine, her weight was 94.5 kg (99th percentile of expected for age and gender), her height was 167 cm (74th percentile), her pulse rate was on the high side (117 breaths/minute) likely due to anxiety and initial blood pressure was 114/88 mmHg (54/97th percentile) - diastolic blood pressure on the high-end expectation for her and and a repeat blood pressure was within normal limit (106/83 mmHg (25/93th percentile). Electrocardiogram (ECG) showed sinus tachycardia, and her lipid profile revealed hypercholesterolaemia (Table 2). Other blood tests like full blood count, kidney function tests, liver function tests, serum prolactin, and fasting blood sugar were normal.

**Table 2:** Results of lipid profile Lipid profile reveals hypercholesterolemia (total cholesterol, non-HDL cholesterol, triglycerides, and LDL cholesterol were higher than normal). HDL: High-density lipoprotein; LDL: low-density lipoprotein.

Parameter	Initial Values	Reference Ranges	Units
Total cholesterol	6.3	3.3-5.2	mmol/L
HDL cholesterol	1.74	1.1-2.6	mmol/L
Non-HDL cholesterol	4.56	4	mmol/L
Cholesterol:HDL ratio	3.6	0-5	
Triglycerides	2.38	<1.7	mmol/L
LDL cholesterol	3.60	3	mmol/L

Treatment and outcome

Four weeks after initiating quetiapine, she was reviewed, and she reported improvement in her mood, sleep, and reduced suicidal thoughts. Her parents described her as happier, and socialising with minimal support. Although quetiapine helped with her mood, getting ready rituals were still distressing and she identified three key areas of improvements: daily personal hygiene, including hair washing, Increased independence in leaving the house, improved self-confidence and reduced fear around routines. Physical observation showed an increase in her weight from 94.5 to 101.2 kg, her diastolic blood pressure was persistently in the 99th percentile (105/91 mmHg and 109/92 mmHg), and her pulse rate on two occasions were 114 and 103 breaths/minute. Despite an increase in weight and elevated diastolic blood pressure, she was keen to try an increase in quetiapine but later opted for a modified release formulation to use only at night because she was feeling tired in the morning after taking quetiapine.

At a three-month review following initiation of quetiapine, she reported daytime sedation, which impacted adherence. She, however, maintained previously highlighted improvements including engagement with home tuition and getting ready rituals becoming less relevant. 

Whilst she did not fully appreciate these improvements, her parents strongly believed that quetiapine contributed significantly to them. Her weight had stabilised at 100.8 kg, and her blood pressure was within normal limits. We compared home readings (blood pressure 115/63 mmHg, pulse rate 93 bpm) with clinic readings (blood pressure 100/87 mmHg, pulse rate 115 bpm), highlighting anxiety as a possible explanation for the elevated pulse rate observed in clinic. Lipid profile and blood glucose were also repeated and, while cholesterol levels remained elevated (Table 3), they were lower than those recorded prior to starting quetiapine (Table 1). Her blood glucose remained within normal limits.

**Table 3:** Results of lipid profile after being on quetiapine for three months Lipid profile reveals hypercholesterolemia (total cholesterol, non-HDL cholesterol, and LDL cholesterol were higher than normal). HDL: High-density lipoprotein; LDL: low-density lipoprotein.

Parameter	Initial Values	Reference Ranges	Units
Total cholesterol	5.9	3.3-5.2	mmol/L
HDL cholesterol	1.73	1.1-2.6	mmol/L
Non-HDL cholesterol	4.17	4	mmol/L
Cholesterol:HDL ratio	3.4	0-5	
Triglycerides	1.52	<1.7	mmol/L
LDL cholesterol	3.56	3	mmol/L

While there are some improvements in her mental health, such as better daily functioning, reduction in the duration of rituals, improved social engagement, starting home schooling, poor adherence to quetiapine leaves room for questions as to whether improvements are due to quetiapine augmentation alone or also related to developmental maturity, increased structure, or improved environmental factors. OCD typically requires consistent treatment for sustained remission [1]. It is unclear whether the observed progress is sustainable without quetiapine; however, the medication may have helped establish early emotional and behavioural stability. Therefore, a collaborative decision to reduce the dose of quetiapine to 12.5 mg at night was made to explore long-term benefit while reducing the risk of side effects.

During this period, there was no ongoing active non-pharmacological intervention. She had three psychological review sessions with the children and young people’s national and specialist OCD and BDD service and was carefully considered for completing the remaining six ASD-adapted CBT sessions. The young person shared that she found the structured nature of ERP and therapy tasks difficult and felt resuming therapy under the same model would likely be unbeneficial. Some of her repetitive behaviours (‘having to do things until it is just right’) was reformulated as likely self-regulatory in the context of ASD and sensory sensitivities. A more flexible approach such as an occupational therapy (OT) input was proposed for consideration.

A referral was made to the service for complex autism and neurodevelopmental disorder (SCAAND) who reviewed her case and made a robust recommendation for further ASD-informed support. Occupational therapy input was planned for exploration through her Education Health Care Plan (EHCP) and she was transitioned to Adult Mental Health Service. 

## Discussion

Methodology

This case report was prepared using clinical information obtained through direct clinical contact with the patient and her family, reviewing past clinical notes and letters from specialist services. 

Written informed consent was obtained from the patient and her parents for the publication of this case report and the clinical information was anonymised.

We conducted a search to identify the existing literature on quetiapine augmentation for OCD. The following electronic databases were searched from inception to December 2025: Ovid Medical Literature Analysis and Retrieval System Online (MEDLINE), Ovid Excerpta Medica Database (Embase), and Ovid Emcare. No restrictions on language were imposed. Key search terms for database searching included: ("obsessive-compulsive disorder" OR OCD) AND (quetiapine) AND (SSRI OR "selective serotonin reuptake inhibitor" OR sertraline OR fluoxetine OR citalopram OR escitalopram OR fluvoxamine OR paroxetine) AND (augmentation OR adjunctive OR "add-on" OR "combination therapy") AND (child OR adolescent OR paediatric OR pediatric OR youth OR "young person" OR teen). 

Search results were downloaded, and duplicate citations was expunged. Titles and abstracts were screened against the inclusion criteria (<18 years, primary OCD, quetiapine+SSRI augmentation, clinical outcomes reported). Four randomised controlled trials, three retrospective studies, one systematic review, and three narrative reviews were retained for literature review and discussion.

Analysis

OCD is a chronic mental health condition, often resulting in significant impairment in daily functioning, especially when complicated with comorbid neurodevelopmental disorders such as ASD. There could be an interplay between these two conditions such as repetitive patterns of behaviour, demand avoidance [5] and difficulties with attention, which can impact engagement in traditional therapeutic approaches such as exposure and response prevention (ERP) [6].

The prevalence of OCD is reported to be 1%-3% in young people with significant impairment affecting the young person and their family [7].

ASD is also frequently found in association with psychiatric disorders. In a systematic review, Van Steensel et al. indicated that 17.4% of adolescents had co-morbid OCD [8]. Due to the prevalence of co-occurrence as well as phenotypic overlap, it is only expected that clinicians will encounter the presentation of repetitive behaviour that does not fit nicely into one diagnosis posing a diagnostic and treatment challenge [9].

These restricted and repetitive behaviours and interests (RRBIs) and obsessive-compulsive behaviours are somewhat similar, e.g., repetition of verbal information like a line from a movie script in autism or repetition of chanting in OCD [9]. In our case, the repetitive behaviour of the patient was initially developed within the framework of OCD as she possessed a full understanding of the ineffectual behaviour bringing her shame and other negative beliefs about herself [10]. Nevertheless, both the mother and the patient recognised that some rituals also provided sensory regulation, which underscores the need for moving beyond a strictly OCD-focused formulation. 

The NICE guideline recommends psychological intervention in the first instance for children and young people with OCD with moderate to severe functional impairment and when they are unable to engage in CBT with ERP or if there has been no adequate response, SSRIs may be considered with careful monitoring. In cases of comorbid conditions, additional or alternative interventions should be considered [1].

The NICE guideline was clear that if treatment with an SSRI in combination with psychological intervention involving the family or carers is unsuccessful or poorly tolerated due to side effects, the use of another SSRI or clomipramine may be considered [1].

Sertraline or fluvoxamine is licensed for OCD in children and is generally considered first line except in cases of comorbid depression where fluoxetine could be considered [1,2]. Some studies have reported that sertraline and fluoxetine are equally effective, but fluvoxamine may be somewhat less so [11]. 

It is well-known that there may be difficulties with engagement with CBT as in the cases of poor insight, learning disabilities, comorbid ASD, and ADHD [2]. In a lot of cases, adaptations are usually required for young people with ASD such as a much slower pace, use of visual aid, and more family involvement. Despite such adjustments, involvement might still be challenging, especially when repetitive activity is being used for a self-regulatory function within the context of ASD. In our case, the patient struggled with regular attendance, demand avoidance, and adherence to ERP tasks. In these instances, medication alone with regular review may be the only available therapeutic option.

Research has shown that about three-quarters of children respond adequately to one SSRI at the maximum tolerated dose for at least 12 weeks in combination with CBT and ERP. It is recommended that roughly a quarter of those who do not respond, following clarifying compliance and a reassessment, should have a trial of at least one other SSRI as approximately 40% of this group of patients respond better to a second SSRI [2].

Clinical features of OCD that may suggest poorer response to SSRI treatment include early age of onset, severity of illness, duration of untreated illness, and the presence of symmetry/ordering or hoarding-related symptoms [12].

If response is poor following a trial of two SSRIs, a recommendation for a referral to a specialist centre is made for the consideration of clomipramine and/or ‘off-label’ augmentation with a low-dose risperidone or aripiprazole [2]. The rationale for using a medication with a different mechanism of action is that it may be beneficial to those with poor response to two adequate trials of SSRI though tolerability and adherence are frequent barriers [13].

Bloch et al., in a systematic review of antipsychotic augmentation of treatment refractory OCD, reported that there is sufficient evidence supporting the efficacy of haloperidol and risperidone while the efficacy of other antipsychotics like quetiapine and olanzapine is unclear [14]. In a meta-analysis of 14 double-blind randomised control trials (RCT), Dold et al. reported that aripiprazole, haloperidol, and risperidone were significantly more effective than quetiapine, olanzapine, and paliperidone compared to placebo [15].

Unfortunately, only one-third of treatment-resistant adult OCD had a notable response to this augmentation regimen. The evidence would therefore suggest restraint in using augmentation for packages of OCD treatment in adolescents and children. A six-week low-dose anti-psychotic augmentation trial would be sufficient to assess efficacy. It should be discontinued in case of no response [2].

Our literature review showed that quetiapine has been attempted for SSRI augmentation with mixed results [16-19]. In a randomised, double-blind, placebo-controlled study of quetiapine augmentation in non-responders to SSRI for 12 weeks, quetiapine administered at a mean daily dose of 169 mg failed to be significantly superior to placebo after six weeks of trial [18]. Another double-blind randomized placebo-controlled trial of augmentation of quetiapine in SSRI-resistant OCD to the maximum dose of 300 mg/day was superior to placebo [19]. Both trials reported quetiapine to be tolerated well with sedation as the most common side effect [18,19].

In this case, quetiapine was introduced primarily to augment SSRI response and improve mood and sleep. While sedation limited adherence, both patient and parent observed clinical benefit, suggesting that even low-dose augmentation may have facilitated emotional stabilization. 

Adherence to treatment was an overriding concern. This implies that ongoing psychoeducation for compliance with medication, especially as adolescents seek greater control of their treatment, is required. Benefit-risk decision-making collaboration, autonomy framing of medication choices, and facilitation of adolescent need for control may reduce demand avoidance and increase long-term involvement.

The hallmark of this case is the challenge that OCD with comorbid neurodevelopmental conditions in young people poses to clinicians and the need for a collaborative, multidisciplinary treatment plan. Coordinated transition planning involving all services in the young person’s care, occupational therapy, and educational support is particularly relevant for young people with neurodevelopmental comorbidities where there may be difficulty accessing services, lack of care coordination, and shared decision making [20]. In this case, referral to specialist adult OCD services and occupational therapy via her EHCP was discussed as part of long-term planning.

Limitations

We acknowledge the absence of standardised symptoms scales (e.g. Children’s Yale‑Brown Obsessive Compulsive Scale (CY-BOCS), a clinician‑rated, semi‑structured tool used to assess the severity of obsessive and compulsive symptoms in children and adolescents) and therefore the dependence on subjective patient and caregiver reports. Due to symptom severity at time, it was not possible to complete a CY-BOCS.

There was difficulty in assigning clinical improvement solely to quetiapine augmentation due to contextual factors (e.g. inconsistent adherence, natural course of the illness, and improved sleep).

## Conclusions

This case illustrates the challenges associated with psychological and pharmacological management of OCD in an adolescent exhibiting poor medication tolerability and comorbid neurodevelopmental conditions. Currently, evidence supporting the use of pharmacogenetic testing in child and adolescent populations is limited, especially for treatment-refractory OCD. Additionally, there is a lack of substantial information regarding the efficacy and side-effect profiles of commonly employed pharmacological enhancement approaches in children and adolescents. In the absence of OCD-specific evidence, this case required careful extrapolation from other psychiatric diagnoses, highlighting the need for research aimed at supporting evidence-based and individualised strategies in complex cases of OCD in children and adolescents.
